# Roles of peptidyl prolyl isomerase Pin1 in viral propagation

**DOI:** 10.3389/fcell.2022.1005325

**Published:** 2022-10-25

**Authors:** Machi Kanna, Yusuke Nakatsu, Takeshi Yamamotoya, Jeffrey Encinas, Hisanaka Ito, Takayoshi Okabe, Tomoichiro Asano, Takemasa Sakaguchi

**Affiliations:** ^1^ Department of Biomedical Chemistry, Graduate School of Biomedical Sciences, Hiroshima University, Hiroshima City, Japan; ^2^ Amenis Bioscience Inc., Seoul, Korea; ^3^ School of Life Sciences, Tokyo University of Pharmacy and Life Sciences, Hachioji, Japan; ^4^ Drug Discovery Initiative, Graduate School of Pharmaceutical Sciences, The University of Tokyo, Bunkyo-ku, Japan; ^5^ Department of Virology, Graduate School of Biomedical Sciences, Hiroshima University, Hiroshima City, Japan

**Keywords:** PPIases, Pin1, virus, human immunodeficiency virus, hepatitis B virus

## Abstract

Peptidyl-prolyl isomerase (PPIase) is a unique enzyme that promotes cis-trans isomerization of a proline residue of a target protein. Peptidyl-prolyl cis-trans isomerase NIMA (never in mitosis A)-interacting 1 (Pin1) is a PPIase that binds to the pSer/pThr-Pro motif of target proteins and isomerizes their prolines. Pin1 has been reported to be involved in cancer development, obesity, aging, and Alzheimer’s disease and has been shown to promote the growth of several viruses including SARS-CoV-2. Pin1 enhances the efficiency of viral infection by promoting uncoating and integration of the human immunodeficiency virus. It has also been shown that Pin1 interacts with hepatitis B virus proteins and participates in viral replication. Furthermore, Pin1 promotes not only viral proliferation but also the progression of virus-induced tumorigenesis. In this review, we focus on the effects of Pin1 on the proliferation of various viruses and discuss the underlying molecular mechanisms.

## Introduction

Peptidyl prolyl isomerase (PPIase) is a unique enzyme that catalyzes the cis-trans isomerization of proline residues, thereby controlling protein folding and stability. Isomerization of proteins by PPIase always occurs at the amide bond of the proline residue. Proline can be both cis- and trans-isomerized because of the relatively weak double bond created by the N lone pair (Matena et al., 2018). The rotational barrier for this isomerization is about 80 kJ mol^−1^ (Schutkowski et al., 1998). The energy barrier for cis-to-trans is lower than that for trans-to-cis (Scherer et al., 1998; Greenwood et al., 2011; Matena et al., 2018), so proline tends to be trans.

### Classification of PPIase

PPIases recognize X (amino acid residue)-proline sequences, where X is different for different PPIase types (Kern et al., 1995; Andreotti, 2003; Alderson et al., 2018). PPIases are basically divided into three enzyme groups: 1) cyclophilin, 2) FKBP (FK506-binding protein), and 3) parvulin.

Cyclophilin group enzymes are the most well-studied as antiviral agents. The structure of human cyclophilin A (hCypA) has a large PPIase domain in the middle and recognizes polypeptides with Ala-Pro or Val-Pro (Dugave and Demange, 2003). Notably, cyclosporin A (CsA), an inhibitor of hCypA, is used as an antiviral drug to treat infections caused by human immunodeficiency virus-1 (HIV-1) and hepatitis C virus (HCV) (Ryffel et al., 1991; Han et al., 2022; Mamatis et al., 2022).

FKBP has a single PPIase domain and preferentially interacts with the Leu-Pro site (Dugave and Demange, 2003). In general, FKBP is abundantly and ubiquitously expressed in many tissues (Cao and Konsolaki, 2011). FK506, an inhibitor of FKBP, is widely used as an immunosuppressant (Harding et al., 1989). FK506 has been used for HCV patients after liver transplantation, together with CyA, a PPIase inhibitor with HCV growth-inhibitory activity (Liu et al., 2014).

Parvulin was initially discovered in *E. coli* as a distinct PPIase that was not inhibited by CsA or FK506 (Rahfeld et al., 1994). Later, Essential in Yeast 1 (ESS1) was identified as a PPIase of the same parvulin family (Hani et al., 1995). Human Pin1 has a structure similar to that of ESS1 and was likewise identified in 1996 as a PPIase of the parvulin family (Lu et al., 1996).

### Function of Pin1 in the cell

Pin1 interacts with NIMA and is essential for mitosis promotion (Lu et al., 1996). Binding to target proteins also requires phosphorylation of serine or threonine before proline (pSer/pThr-Pro motif), and the N-terminal WW domain of Pin1 recognizes the phosphorylation site. Although phosphorylation of serine normally slows the rate of isomerization of the immediate proline-amide bond (Schutkowski et al., 1998), Pin1 binds to this phosphorylated serine and promotes the isomerization reaction of the proline-amide bond by the C-terminal PPIase domain.

Pin1 is expressed in numerous organs including the lung, liver, stomach, brain, heart, kidney, and spleen (Ryo et al., 2001) and may be a key factor associated with many metabolic pathways; Pin1 knockout (KO) mice are born and live to an old age (Fujimori et al., 1999) but develop weight loss, osteoporosis, skin atrophy, retinal degeneration, genital atrophy, and impaired motor coordination (Fujimori et al., 1999).

Pin1 is upregulated in malignant tumors, hepatitis, and metabolic syndrome-related diseases such as obesity and diabetes and has been reported to be involved in promoting the pathogenesis of these diseases (Liou et al., 2003; Rustighi et al., 2017; Inoue et al., 2019). On the other hand, Pin1 KO mice exhibit tau aggregation in the brain and a phenotype of behavioral abnormalities (Lu et al., 1999). This indicates that Pin1 deficiency is a risk factor for Alzheimer’s disease, and Pin1 may work in the direction of disease suppression.

### Involvement of Pin1 in viral replication

Pin1 has been shown to be involved in viral replication. The following is a virus-by-virus summary of Pin1’s involvement in viral replication.

### SARS-CoV-2

Severe acute respiratory syndrome coronavirus-2 (SARS-CoV-2), which has caused a global pandemic since 2019 and has greatly affected our lives, belongs to the family Coronaviridae. Coronaviruses have positive-sense single-stranded RNA genomes, and many species have been reported in animals. In humans, four viruses that cause the common cold and SARS-CoV, Mideast Respiratory Syndrome (MERS) CoV, and SARS-CoV-2 that cause severe pneumonia are known (Perlman and Masters, 2021). Among these, the growth of feline coronavirus was reported to be enhanced by Pin1 (Tanaka et al., 2016). Our group showed that SARS-CoV-2 growth was inhibited by Pin1 knockdown or Pin1 inhibitors (Yamamotoya et al., 2021). In doing so, viral transcription was inhibited, indicating that RNA synthesis of SARS-CoV-2 is likely to be promoted by Pin1 (Yamamotoya et al., 2021).

On the other hand, unlike HIV-1 and other viruses, the proliferation of SARS-CoV-2 was not inhibited by either CsA or FK506 (unpublished observations). Therefore, neither hCypA nor FKBP is thought to be involved in the proliferation of SARS-CoV-2.

It was also reported that Pin1 binds to the phosphorylated Ser79-Pro80 sequence of the N protein (Ino et al., 2022). Interestingly, this binding site of the N protein was specific to SARS-CoV-2 and was not found in closely related viruses such as SARS-CoV and MERS-CoV. Further investigation is needed to determine whether the Ser-Pro sequence of this N protein is involved in the growth of SARS-CoV-2.

### Human immunodeficiency virus-1 (HIV-1)

**FIGURE 1:**
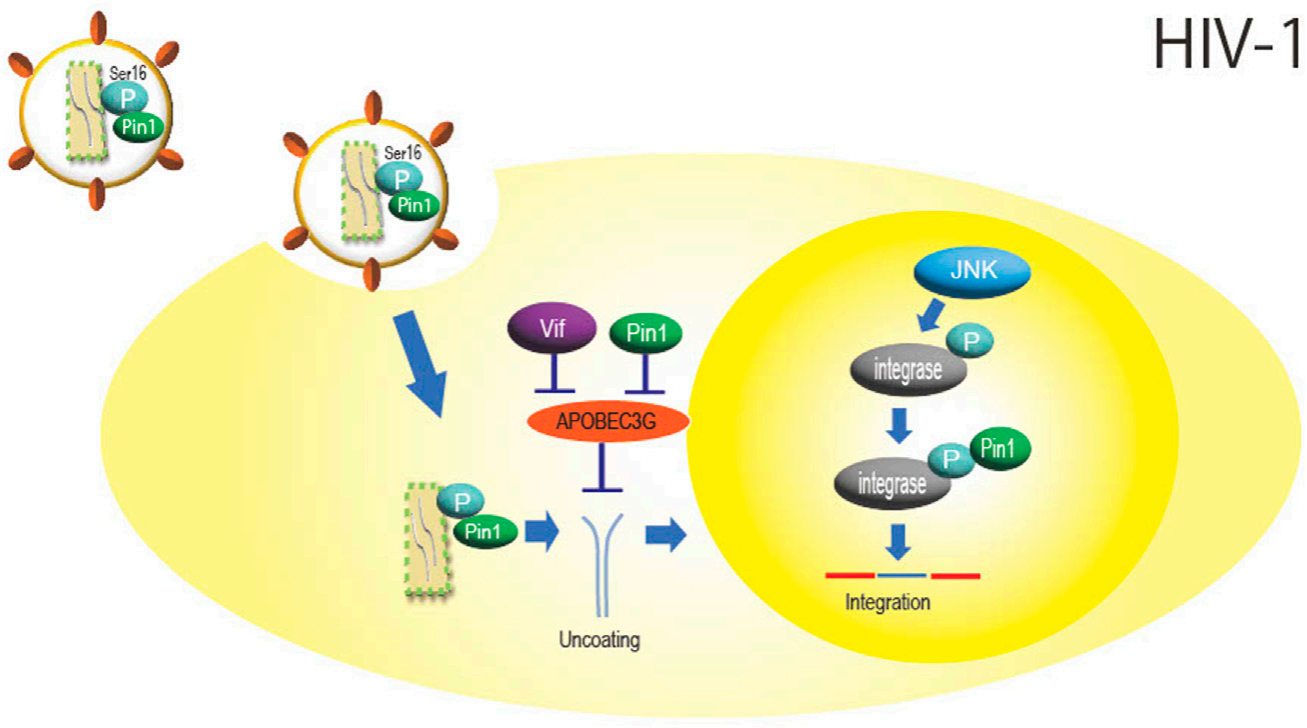
Role of Pin1 in HIV-1 infection. Inside the HIV-1 particle, Pin1 is thought to bind with the capsid protein at phosphorylated Ser16-Pro17, which promotes nucleocapsid uncoating. APOBEC3G inhibits HIV-1 replication by mutating the viral genome, but Pin1 binds to APOBEC3G and inhibits this function independently of Vif. Integrase is phosphorylated by JNK in the nucleus, and Pin1 binds to this site to enhance integration efficiency.

HIV-1 belongs to the family *Retroviridae* and is a virus that causes acquired immunodeficiency syndrome. The Ser residue in the Ser16-Pro17 sequence of the HIV-1 capsid protein is phosphorylated by extracellular signal-regulated kinase 2 (ERK) (Courcelles et al., 2013), and Pin1 binds to this phosphorylation site and may aid HIV-1 infection by promoting uncoating (Figure 1) (Misumi et al., 2010; Dochi et al., 2014) Since this phosphorylation occurs inside the HIV-1 particle before entering the cell (Dochi et al., 2014) and since Pin1 itself is also incorporated into the HIV-1 virion (Ott et al., 2000), it is possible that isomerization of the capsid protein by Pin1 also already occurs within the virus particle.

APOBEC3G, a DNA cytidine deaminase constitutively expressed in human cells, is a protein involved in innate immunity that is incorporated into HIV-1 particles during particle formation and inhibits infection by impairing HIV-1 genome replication as the progeny virus infects new cells. Pin1 binds to APOBEC3G and inhibits its action and incorporation into viral particles (Watashi et al., 2008). Although HIV-1 is known to degrade APOBEC3G *via* the accessory protein Vif (Cullen, 2006), it is thought that Pin1 is also involved in Vif-independent counteraction against APOBEC3G function (Figure 1).

In addition, virus integrase is phosphorylated at the Ser57-Pro58 site by intranuclear c-Jun N-terminal kinase (JNK), and Pin1 binds to this phosphorylation site to enhance integrase stability (Manganaro et al., 2010). JNK activation is mediated by NF-kB signaling, which may facilitate HIV integration (Figure 1) (Saleh et al., 2016). As described above, HIV-1 is thought to efficiently multiply by utilizing Pin1 in several steps of viral replication, such as uncoating and integration.

### Hepatitis B virus (HBV)

Hepatitis B virus (HBV), a member of the family *Hepadnaviridae* that causes acute and chronic hepatitis, cirrhosis, and liver cancer, has a circular double-stranded DNA genome with a gap and significantly overlapped coding regions (Seeger and Mason, 2015). Pin1 is also involved in HBV replication, since Pin1 knockdown results in reduced amounts of HBV cccDNA and pgRNA (pregenome RNA) (Zhou et al., 2021).

**FIGURE 2:**
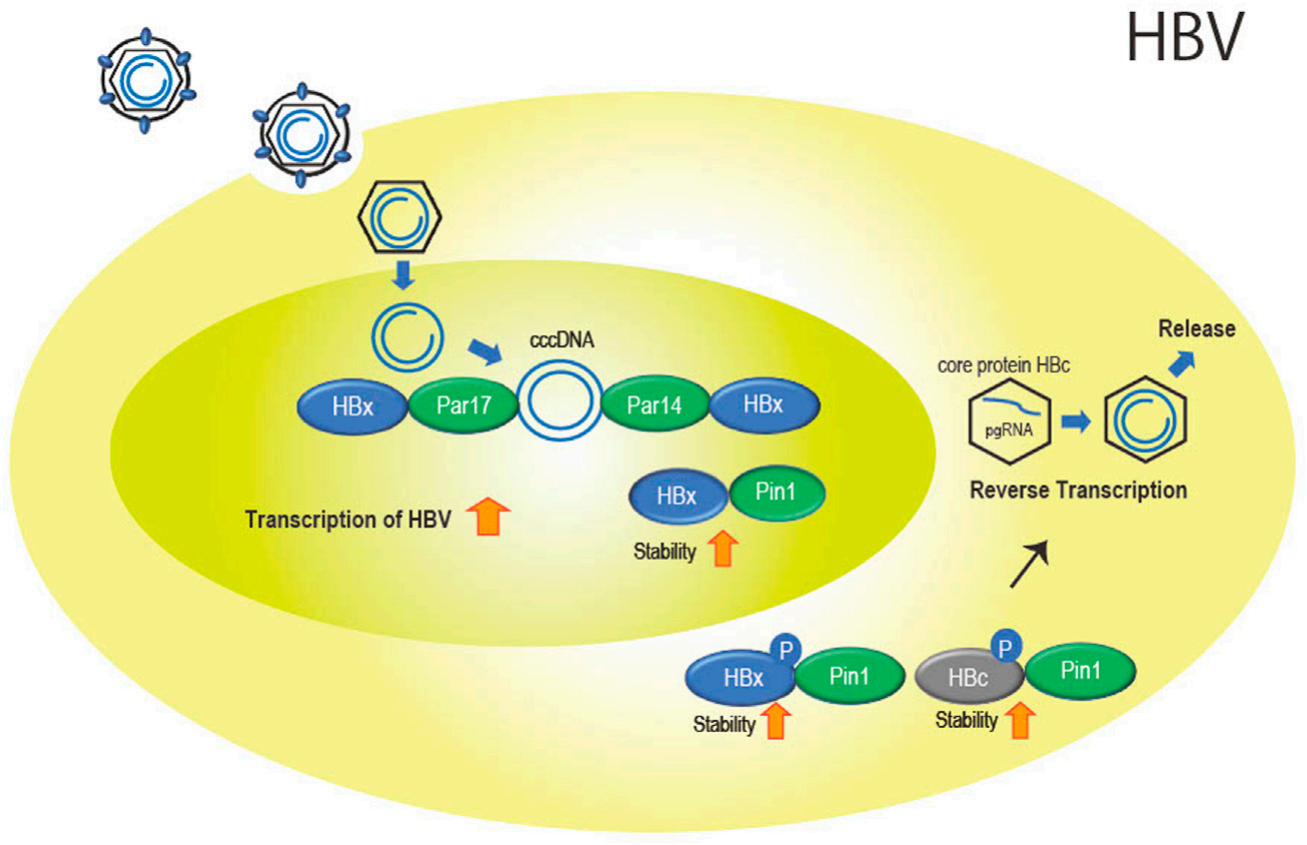
Role of the parvulin family in HBV infection. Par14 and Par17 bind to both cccDNA and HBx in the nucleus. This is thought to promote cccDNA replication and transcription of viral genes with stabilized HBx with Pin1 binding. In the cytoplasm, Pin1 also binds to HBx and HBc to promote nucleocapsid formation and reverse transcription as well as particle formation.

Specifically involved with Pin1 is HBV protein X (HBx), encoded by the X gene, which is a multifunctional protein involved in promoting viral replication (Xu et al., 2002) and the release of viral particles from cells (Reifenberg et al., 2002). Pang et al. (2007) showed that Pin1 and HBx interact directly in the cytoplasm and nucleus. The interaction stabilizes HBx and increases its function (Figure 2). The Pin1-binding site, the Ser41-Pro42 sequence of HBx, is conserved in most types of viral strains (Kidd-Ljunggren et al., 1995; Datta et al., 2007), suggesting that stabilization of HBx by Pin1 is essential for HBV replication.

Parvin14 (Par14) (Uchida et al., 1999) which is encoded by the Pin 4 gene and belongs to the parvulin family as does Pin1, and the isoform Parvin17 (Par17) also interact at Ser41-Pro42 in HBx (Saeed et al., 2019). In addition, cccDNA binds to Par14 and Par17 and serves as a bridge between cccDNA and HBx (Saeed et al., 2019) (Figure 2).

In addition to HBx, Pin1 binds to phosphorylated Thr160-Pro161 and Ser162-Pro163 of the core protein (HBc) involved in formation of the nucleocapsid of HBV by stabilizing HBc (Figure 2). The binding of Pin1 to the phosphorylation sites allows HBc to escape from degradation in lysosomes (Nishi et al., 2020).

Furthermore, Pin1 may act as a trigger factor for cancer progression in tumorigenesis after HBV infection. Overexpression of HBx and Pin1 in the non-tumorigenic human hepatoma cell line MIHA was found to result in the formation of large tumors in an animal transplantation model (Pang et al., 2007). This suggests that Pin1 and HBx promote the growth of hepatocellular carcinoma (HCC). Interestingly, even in Pin1-unbound HBx, overexpression of Pin1 in cell lines leads to tumor growth (Pang et al., 2007). Pin1 may also be associated with other pathways of tumorigenesis.

Pin1 has been found to activate 56 oncogenes and inactivate 26 tumor suppressor genes (Yu et al., 2020); since Pin1 is closely related to cell growth, the virus may take advantage of this property to promote its own tumorigenesis.

Since Pin1 is highly expressed in HCC (Pang et al., 2007), Pin1-based HCC therapeutics have been actively investigated (Cheng et al., 2016; Cheng and Tse, 2020). Pin1 siRNA-based drug delivery systems have been tested for treatment of HCC (Zhao et al., 2021). Anticancer agents, including Pin1-specific inhibitors such as juglone (Hennig et al., 1998), all-trans-retinoic acid (ATRA) (Wei et al., 2015), and Sulphopin, which targets the active center (C113) of PPIase as does juglone (Dubiella et al., 2021), are eagerly awaited.

### Pin1 action on other viruses

Tax of human T-lymphotropic virus-1 (HTLV-1) of the family *Retroviridae*, which causes adult T-cell leukemia (ATL) and HTLV-1-associated myelopathy (HAM), causes transcriptional regulation, proliferation, and transformation of virus-infected cells. Tax also activates NF-kB for cell proliferation. Pin1 binds to the phosphorylated Ser160-Pro161 of Tax, stabilizes Tax (Jeong et al., 2009), and promotes proliferation of infected cells with Tax-induced NF-kB activation (Peloponese et al., 2009).

In infection with hepatitis C virus (HCV) of the family *Flaviviridae*, Pin1 interacts with NS5A and NS5B (RNA-dependent RNA polymerase), which are involved in viral RNA synthesis and promote HCV genome replication (Lim et al., 2011).

In cytomegalovirus (CMV) of the family *Herpesviridae*, the genome replicates in the nucleus, resulting in the formation of a nuclear egress complex (NEC) that buds at the nuclear membrane to produce progeny viruses. The lamina around the nuclear membrane, on the other hand, serves as a physical barrier. Viral infection phosphorylates Ser22-Pro23 of the laminin, the main component of the lamina (Lee and Chen, 2010), and Pin1 binds to the phosphorylated site to isomerize the laminin, degrading the lamina structure and allowing viral budding at the nuclear membrane (Milbradt et al., 2016). This is thought to be true for other *Herpesviridae* viruses.

Pin1 has also been shown to bind directly to other CMV proteins, such as the core nuclear egress complex protein pUL50, viral mRNA export factor pUL69, and viral DNA polymerase processivity factor pUL 44, and may regulate multiple steps of viral replication including gene replication in cytomegaloviruses (Schütz et al., 2020).

Rta, which is involved in the switch from latent infection to reactivation in Kaposi’s sarcoma-associated herpesvirus (KSHV)-infected cells, is a proline-rich protein and is highly phosphorylated; Pin1 binding to Rta is thought to promote transcriptional activation and reactivation (Guito et al., 2014). On the other hand, Pin1 inhibits the synthesis of KSHV structural proteins during the late phase of reactivation. Thus, Pin1 is thought to be a bidirectional positive and negative regulator of KSHV reactivation and a molecular timer that controls the progression of KSHV proliferation (Guito et al., 2014).

### Involvement of Pin1 in the immune system related to viral replication

It has been shown that the type 1 interferon system, an innate immunity that mainly acts against viral infection, is negatively regulated by Pin1 (Saitoh et al., 2006). IRF3, a positive regulator of IFN-α/β, is phosphorylated at Ser339 *via* signal transduction induced by duplex RNA; Pin1 binding to the phosphorylation site, Ser339-Pro340, causes IRF3 to be degraded in the proteasome after polyubiquitination, resulting in inhibition of IRF3 activation (Saitoh et al., 2006).

On the other hand, it has been shown that Pin1 binds to phosphorylated IRAK1 upon stimulation of TLR7 and TLR9, activating IRAK and promoting IRF7-mediated interferon 1 secretion (Tun-kyi et al., 2011). Thus, Pin1 has been shown to have both accelerator and brake functions in the innate immune system, but the full, rather than partial, role of Pin1 in immunity needs to be further elucidated.

In T cells, Pin1 has also been implicated in the regulation of T cell-mediated immunity by regulating mRNA stability, apoptosis, and proliferation (Liu et al., 2001; Esnault et al., 2008).

## Summary

Pin1 is ubiquitously distributed in organs and binds to various cellular proteins to regulate biological functions. When viruses infect cells, many viruses promote viral replication. The point of action and mechanism of action differ among viruses, and at least for HIV-1, HBV, and CMV, it has been shown that the same virus acts on multiple targets. If we can develop appropriate Pin1 inhibitors, we may be able to create broad-spectrum antiviral agents that are effective against various viruses.
